# The role of malignancy in the mortality of Stevens-Johnson syndrome and toxic epidermal necrolysis

**DOI:** 10.1186/2045-7022-4-S3-P21

**Published:** 2014-07-18

**Authors:** Jennifer Wu, Yung-Yi Lee, Wen-Hung Chung

**Affiliations:** 1 Chang Gung Memorial Hospital, Dermatology Department, Taiwan; 2 Chang Gung Memorial Hospital, Drug Hypersensitivity Clinical and Research Center, Taiwan

## Background

Stevens-Johnson syndrome (SJS) / Toxic epidermal necrolysis (TEN) is a rare, severe, and occasionally fatal skin reaction caused mainly by medications. The reported mortality associated with TEN varies from 10-70%. SCORTEN, a severity-of-illness score which estimates the mortality in TEN has been developed and validated. Malignancy was considered as 1 of the 7 independent prognostic factors of SCORTEN. However, the studies focused on the associated factors of malignancy in the mortality of SJS/TEN were limited. In this study, we tried to analyze the true role of malignancy in the mortality of SJS/TEN.

## Method

We retrospectively reviewed the medical charts of the patients diagnosed of ve SJS, SJS/TEN overlap syndrome, and TEN in a single tertiary medical center in Taiwan from 2002 to 2012.

## Result

A total of 527 patients of SJS/TEN were included in this study (mean age 50.58 years). 55 patients were found to have malignancies (mean age 65.64 years). The most common malignancy in patients of SJS/TEN was lung cancer (10), followed by malignant lymphoma (8), urothelial carcinoma (6), and hepatocellular carcinoma (5), etc. The mortality was high in the patients with underlying malignancies (29.09%) compared to those without malignancies (9.51%) (OR=4.3127, p value<0.05). The mortality varied in malignancies of different kinds or of different organ systems, however, no statistic significance was identified. The mortality was higher in the patients with malignancies of stage 4 (34.48%) compared to those of stage 0 to 3 (23.81%) without statistic significance. The patients with malignancies who underwent the chemotherapy (50%) seemed to have higher mortality compared to those who did not (22.22%) (OR=3.5, p value=0.057).

## Conclusion

The mortality was significantly higher in patients with underlying malignancies, but varied in different kinds of malignancies and malignancies of different organ systems. The role of malignancy in mortality of SJS/TEN is far more than current all-or-none consideration of SCORTEN. Chemotherapy, advanced stage, and certain kinds of malignancies showed a trend of higher mortality in patients of SJS/TEN with underlying malignancy. Further efforts must be done to clarify the true role of malignancy in the mortality of SJS/TEN.

